# A bibliometric analysis of non-coding RNAs in Parkinson disease: Research hotspots and emerging trends (2013–2022)

**DOI:** 10.1097/MD.0000000000046852

**Published:** 2025-12-19

**Authors:** Lina Zhu, Deng Chen, Xiangxiu Wang, Chengqi He

**Affiliations:** a Rehabilitation Medicine Center and Institute of Rehabilitation Medicine, West China Hospital, Sichuan University, Chengdu, Sichuan Province, China; b Key Laboratory of Rehabilitation Medicine in Sichuan Province, West China Hospital, Sichuan University, Chengdu, Sichuan Province, China; c Department of Neurology, West China Hospital, Sichuan University, Chengdu, Sichuan Province, China.

**Keywords:** CiteSpace, hotspot, non-coding RNA, Parkinson disease, trend, visual analysis

## Abstract

Increasing evidence indicates that non-coding RNAs (ncRNAs) play a crucial role in the pathological processes of Parkinson disease (PD). However, bibliometric research focusing on PD and ncRNAs remains limited. Articles and reviews related to PD and ncRNAs published over the past decade were retrieved from the Web of Science Core Collection. Bibliometric visualization websites and software, including CiteSpace and VOSviewer, were used for the analysis. A total of 1116 studies were identified. Both the number of publications and citations showed an upward trend. China, USA, and Italy were the top 3 most productive countries. Keyword analysis revealed that current research mainly focuses on “alpha-synuclein” and “microRNAs.” However, emerging research topics such as “endoplasmic reticulum stress,” “inflammation,” and “circular RNAs” could represent frontier directions in this field. Our results suggest that research related to ncRNAs and PD continues to attract attention. This bibliometric analysis may provide valuable insights to help future researchers choose their research directions.

## 1. Introduction

Parkinson disease (PD) is a neurodegenerative disorder characterized by classical motor symptoms, known as “parkinsonism,” and a spectrum of non-motor symptoms.^[1]^ The key pathological features of PD include the progressive loss of dopaminergic neurons in the substantia nigra (SN), the presence of Lewy pathology, and sustained neuroinflammation.^[1]^ The prevalence of PD has been increasing in recent years.^[2]^ As a leading cause of motor disability, PD significantly impairs patients’ quality of life and imposes a severe economic burden.^[3]^

The interaction between environmental factors and genetic background is known to contribute to PD pathogenesis^[4]^; however, the role of epigenetic regulation in this disease is less understood. Accumulating evidence has shown that non-coding RNAs (ncRNAs) are extensively involved in regulating physiological processes in the nervous system and are implicated in the pathogenesis and progression of PD.^[5]^ For instance, a group of long non-coding RNAs (lncRNAs), including metastasis-associated lung adenocarcinoma transcript 1 (MALAT1), H19, and small nucleolar RNA host gene 1, are differentially expressed in PD and may participate in disease progression by influencing synaptogenesis and neuronal apoptosis.^[6]^ Similarly, the abnormal expression of lncRNA-nuclear-enriched abundant transcript 1 (NEAT1) has been identified in both the peripheral blood^[7]^ and the SN of PD patients,^[8]^ where it may exert a neuroprotective effect against drug-induced oxidative stress.^[8]^ More recently, circular RNAs (circRNAs), another class of ncRNAs, have been implicated in PD pathology. For example, circSLC8A1 was found to be upregulated in the SN of PD patients and may be associated with oxidative stress.^[9]^ Additionally, circSNCA has been shown to promote apoptosis and suppress autophagy in cellular models of PD.^[10]^

To date, bibliometric analyses in PD research have primarily addressed general research trends, therapeutic strategies, or broad molecular mechanisms, without dedicated assessment of the rapidly growing literature on ncRNAs in PD. Although a recent bibliometric study analyzed the involvement of microRNAs (miRNAs) in PD from 2014 to 2023, it did not incorporate lncRNAs or circRNAs.^[11]^ In this study, we employed a multi-platform approach – using CiteSpace, VOSviewer, and an online bibliometric tool – to perform the first comprehensive bibliometric mapping focused exclusively on publications linking PD with all major ncRNA categories, including miRNAs, lncRNAs, and circRNAs, over the past decade. This analysis summarizes current research hotspots, identifies leading institutions, and highlights emerging frontiers such as endoplasmic reticulum (ER) stress, inflammation, and circRNA mechanisms in PD, thereby providing valuable insights for future research directions.

## 2. Materials and methods

### 2.1. Data source and search strategy

We conducted an online literature search of the Web of Science Core Collection (WoSCC) on December 22, 2023. The search strategy is described in Table S1 (Supplemental Digital Content, https://links.lww.com/MD/R50). The search parameters were as follows: index: science citation index-expanded; timespan: 2013 to 2022; language: English; document types: article or review.

The exclusion criteria were: articles not substantively relevant to PD, where ncRNAs were only briefly mentioned in the background; publication types such as conference abstracts, editorials, corrections, perspectives, and book reviews; and articles lacking available bibliographic information (title, abstract, and keywords all missing) or retracted publications.

The initial search results were exported as standard files containing all bibliographic fields (e.g., title, abstract, keywords, authors, affiliations, publication year, DOI, and citation counts). Deduplication was first performed automatically using DOI as primary key. If DOI was missing, exact or fuzzy matching based on title (case- and punctuation-insensitive), first author, and publication year was applied, with manual verification when necessary. The deduplication process retained the first exported complete record for traceability, and the number and rationale for excluding duplicates were documented.

The deduplicated records were independently screened by 2 researchers based on title and abstract in a blinded manner. Records that could not be definitively assessed by title/abstract proceeded to full-text evaluation. Both reviewers documented the reasons for inclusion, exclusion, or uncertainty at each stage. Discrepancies were resolved by a third researcher, who made the final decision.

To assess the robustness of the data cleaning and screening process, a random sample of 5 to 10% (or no fewer than 50 articles) of the finally included literature was manually rechecked for bibliographic accuracy and thematic relevance. Additionally, the full texts of the top 20 most-cited articles were reviewed to confirm their primary focus on PD and ncRNA research.

### 2.2. Data analysis

**Figure 1.**
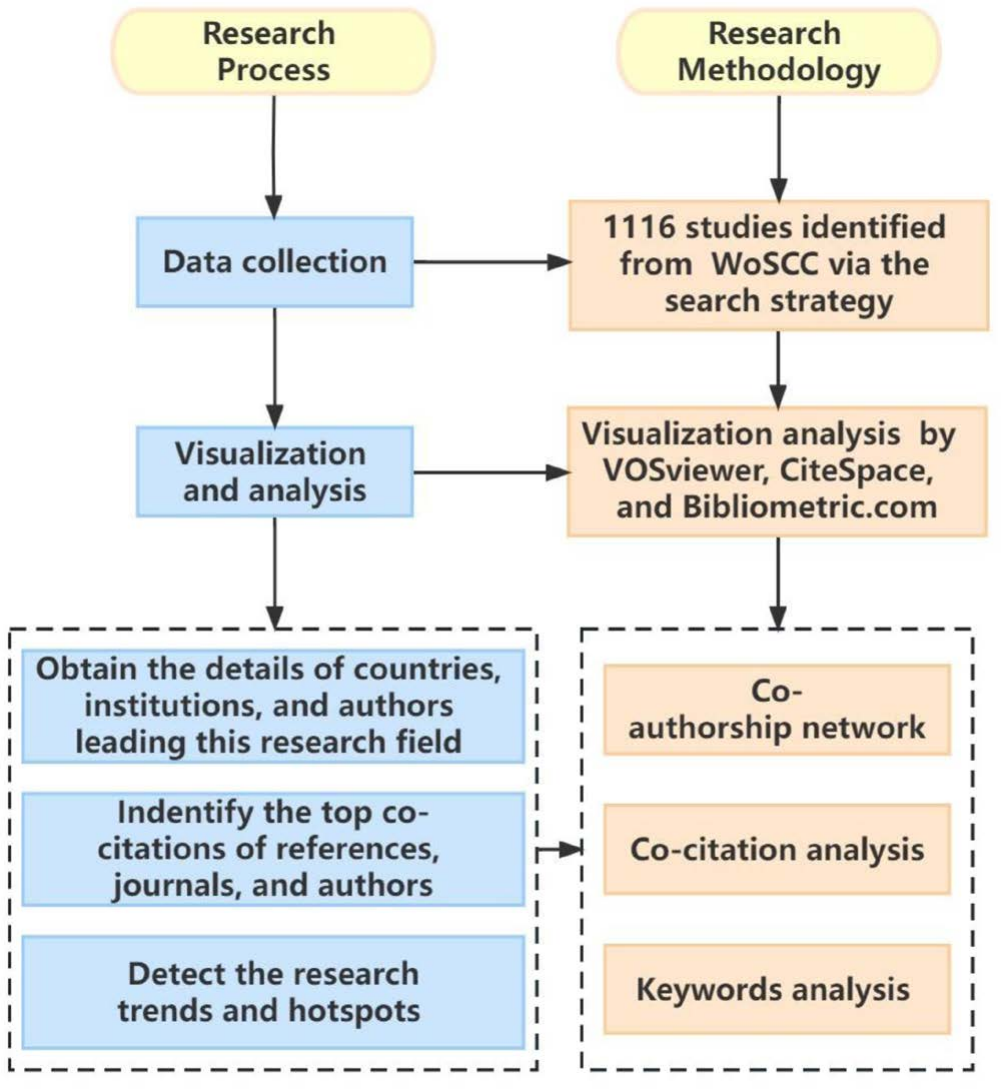
Study flow diagram.

After data extraction from the selected literature, data analysis was performed using the bibliometric visualization website (https://bibliometric.com/) along with specialized software tools, including CiteSpace 6.1.6 (Drexel University, Philadelphia, PA) and VOSviewer 1.6.18 (Leiden University, Van Eck NJ). The overall workflow is summarized in Figure 1.

Co-authorship networks of authors, countries, and institutions were constructed using CiteSpace based on co-occurrence analysis. The bibliometric visualization website was also employed to illustrate collaborative relationships among countries in this research field. In addition, VOSviewer was used to generate co-citation visualizations involving references, journals, and authors. In these maps, different nodes represent distinct elements such as co-cited references, journals, or authors. The size of a node corresponds to the citation frequency, with larger circles indicating higher citation counts. Lines connecting nodes represent co-citation relationships, while the colors of nodes and lines reflect different publication years or clusters.

In the keywords analysis, co-occurrence analysis of keywords by overlay visualization was also performed by VOSviewer. CiteSpace software was used to detect the research trends and hotspots through clustering, and burst analysis of keywords. The parameters in CiteSpace were configured as follows: Time Slicing (2013–2022), Years per Slice (1), Term Source (all selected), Selection Criteria (top N = 50), Pruning (pathfinder, pruning sliced networks), and Visualization (cluster view-static, show merged network). In the resulting graphs, node size is proportional to the frequency of occurrence or citation. The color and thickness of nodes indicate the number of occurrences or citations within specific time periods. A purple outer ring around a node denotes high centrality (≥0.1), signifying key points or turning points in the field.

## 3. Results

### 3.1. Analysis of publication outputs and growth trend

**Figure 2.**
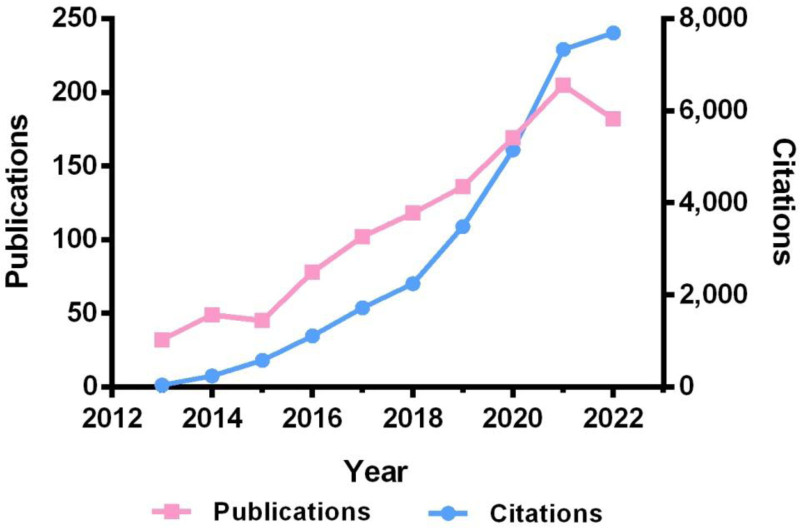
Number of published papers and citations in the field of PD and ncRNAs from 2013 to 2022. ncRNAs = non-coding RNAs, PD = Parkinson disease.

A total of 1116 publications on PD and ncRNAs were identified, comprising 71.4% articles and 28.6% reviews. As shown in Figure 2, the annual publication count in this field has exhibited a steady upward trend over the past decade. Despite minor fluctuations in 2015 (45 publications) and 2022 (182 publications), the number of annual citations surged dramatically from 44 in 2013 to 7690 in 2022.

### 3.2. Co-authorship: countries, institutions, and authors

**Figure 3.**
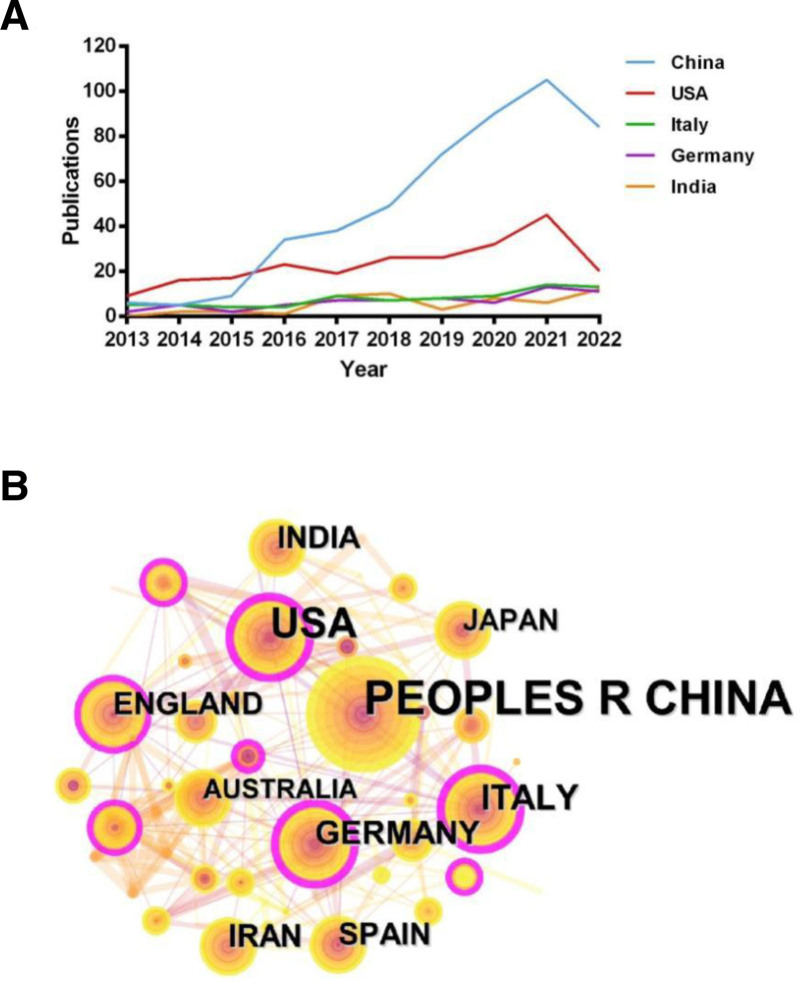
(A) Annual publication trends (2013–2022) of the top 5 countries in Parkinson disease and ncRNA research; (B) Network diagram of countries. ncRNAs = non-coding RNAs.

**Figure 4.**
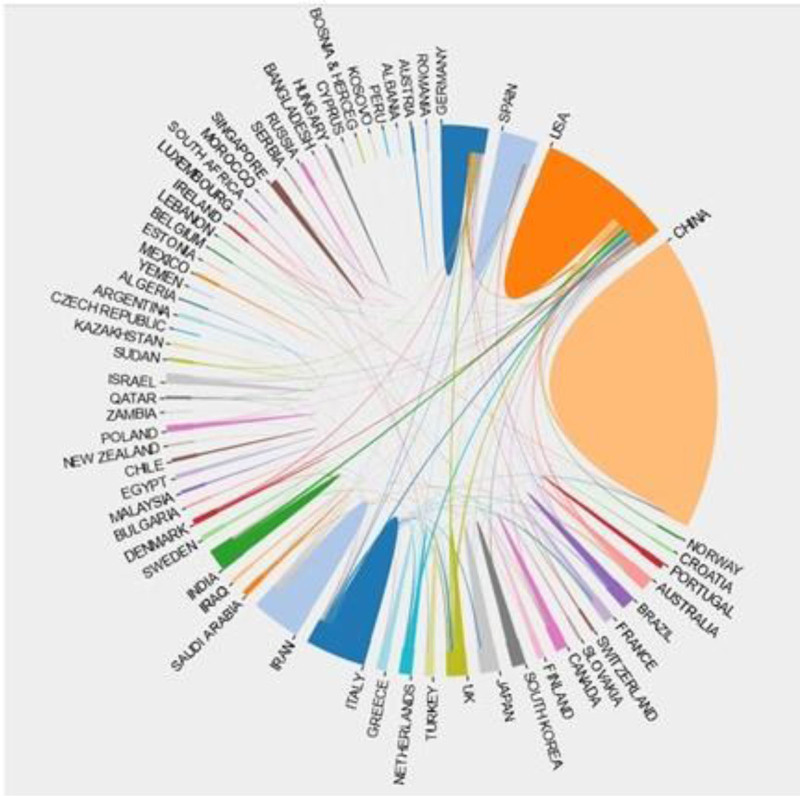
International collaboration network in the field of Parkinson disease and ncRNAs. This chord diagram illustrates research collaborations among different countries in the field of Parkinson disease (PD) and non-coding RNAs (ncRNAs). The thickness of the chords represents the intensity of collaboration, while different colors distinguish the countries. China and the USA occupy central positions in this network, maintaining strong collaborative links with multiple countries.

From 2013 to 2022, researchers from 69 countries contributed to the field of PD and ncRNAs. China and the USA were the most productive, publishing 492 (44.09%) and 232 (20.79%) papers, respectively (Table 1), together accounting for over half of all publications. Details of the top 5 countries, including their citation counts and H-index, are presented in Table 2, and their annual publication trends are shown in Figure 3A. Regarding centrality, Germany (0.38), the USA (0.33), and Italy (0.22) ranked highest (Table 3). Countries with high centrality are highlighted by a purple outer ring in the network map (Fig. 3B). Furthermore, the USA demonstrated the highest level of international collaboration (Fig. 4).

**Table 1:** The top 10 active countries, institutions and authors.

Rank	Country	Records	Institution	Records	Author	Records
1	China	492	Zhengzhou University	22	Ghafouri-fard, Soudeh	9
2	USA	232	China Medical University	19	Ghaedi, Kamran	9
3	Italy	78	Capital Medical University	18	Taheri, Mohammad	8
4	Germany	66	Nanjing Medical University	17	Soreq, Hermona	7
5	India	53	Nanchang University	16	Liu, Wei	7
6	England	46	Islamic Azad University	14	Zhang, Shizhong	6
7	Iran	44	Southern Medical University	14	Peymani, Maryam	6
8	Spain	43	Dalian Medical University	13	Lu, Guohui	6
9	Japan	35	Jilin University	13	Chen, Yong	6
10	Australia	33	Henan University	12	Lingor, Paul	5

Rankings are based on the number of publications, with China, Zhengzhou University, and Ghafouri-fard Soudeh leading in their respective categories.

**Table 2:** Top 5 countries with the most publications.

Rank	Country	Citations	Citations (without self-citations)	Average citations	H-index
1	China	10,596	8713	21.54	52
2	USA	9271	8924	39.79	55
3	Italy	2955	2882	37.88	30
4	Germany	2323	2287	35.2	28
5	India	1446	1414	27.28	22

The details of the top 5 countries with the most publications, including total citations, citations excluding self-citations, average citations per paper, and H-index, with the USA leading in citation impact and China leading in publication volume.

**Table 3:** The top 10 countries and institutions with high centrality value.

Rank	Country	Centrality	Institution	Centrality
1	Germany	0.38	German Center for Neurodegenerative Diseases	0.15
2	USA	0.33	University of Freiburg	0.15
3	Italy	0.22	Banner Sun Health Research Institute	0.15
4	England	0.15	University Medical Center Göttingen	0.14
5	France	0.15	Stanford University	0.13
6	Netherlands	0.15	Dalian Medical University	0.13
7	Denmark	0.15	CNR	0.13
8	IRAQ	0.11	Aarhus University	0.13
9	India	0.10	China Medical University	0.12
10	Spain	0.10	Boston University	0.12

The data on the top 10 countries and institutions with the highest centrality values, indicating their pivotal roles in international collaboration networks, with Germany and the German Center for Neurodegenerative Diseases (DZNE) ranking first in their respective categories.

The top 10 most productive institutions in this field are listed in Table 1, most of which are located in China. Zhengzhou University ranked first with 22 publications, followed by China Medical University (19 publications) and Capital Medical University (18 publications). When ranked by centrality (Table 3), however, the German Center for Neurodegenerative Diseases, University of Freiburg, and Banner Sun Health Research Institute emerged as the leading institutions. As shown in Table 1, the most prolific authors were Ghafouri-fard S (9 publications), Ghaedi K (9 publications), and Taheri M (8 publications).

### 3.3. Co-citation: references, journals, and authors

VOSviewer was employed to visualize the co-citation networks of references, journals, and authors in this field. According to a previous study,^[11]^ co-cited references are those cited simultaneously by 2 or more publications, while co-cited authors and journals are derived from these co-cited references. The top 10 entries for each co-citation category are detailed in Table 4.

**Table 4:** The top 10 co-citations, including references, journals, and authors.

Rank	References	Citations	Journals	Citations	Authors	Citations
1	A MicroRNA feedback circuit in midbrain dopamine neurons	194	PLoS One	2266	Kim J	264
2	Repression of alpha-synuclein expression and toxicity by microRNA-7	181	P Natl Acad Sci USA	2203	Junn E	249
3	MicroRNA profiling of Parkinson disease brains identifies early downregulation of miR-34b/c which modulate mitochondrial function	164	Nature	1847	Hebert SS	211
4	Post-transcriptional regulation of alpha-synuclein expression by mir-7 and mir-153	159	J Boil Chem	1647	Doxakis E	177
5	MicroRNA-205 regulates the expression of Parkinson disease-related leucine-rich repeat kinase 2 protein	110	Science	1531	Bartel DP	177
6	Identification of blood microRNAs associated to Parkinsonĭs disease	101	J Neurosci	1502	Miñones-Moyano E	173
7	Parkinson disease	100	Cell	1416	Soreq L	157
8	Convergence of miRNA expression profiling, α-synuclein interacton and GWAS in Parkinson disease	97	Nucleic Acids Res	1268	Zhang Y	153
9	Altered microRNA profiles in cerebrospinal fluid exosome in Parkinson disease and Alzheimer disease	97	Hum Mol Genet	1167	Alvarez-Erviti L	145
10	Plasma-based circulating MicroRNA biomarkers for Parkinson disease	88	Movement Disorders	1061	Cardo IF	128

The most co-cited reference, journal, and author in the field’s top 10 are “A microRNA feedback circuit in midbrain dopamine neurons,” PLoS One, and Kim J, respectively.

miRNA = microRNA.

**Figure 5.**
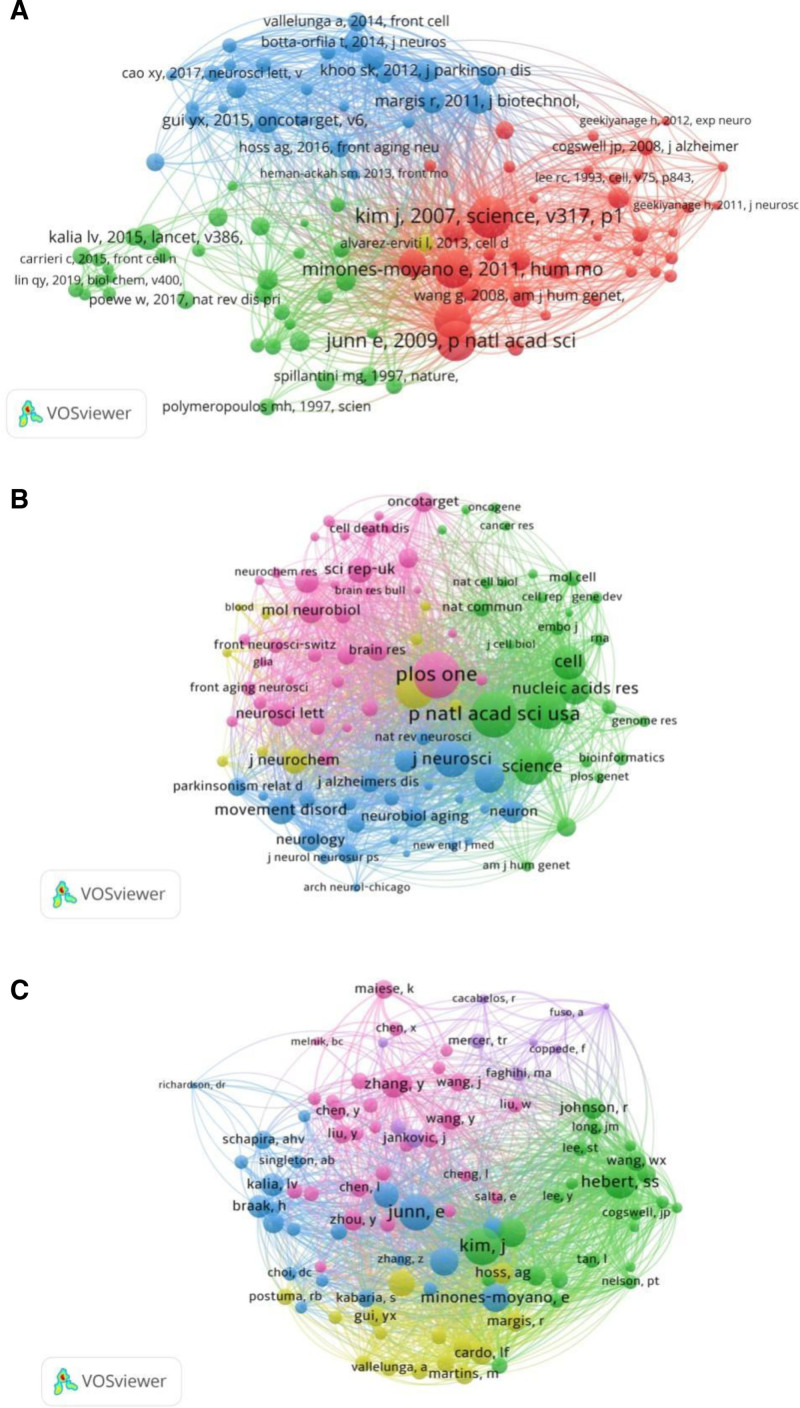
Reference co-citation analysis. (A) Network visualization of the top 100 most co-cited references (total references cited: 54,286; highest co-citation count: 194; total link strength: 2339). (B) Co-cited journal network. PLoS One ranked first (cited 2266 times), followed by PNAS (2203 times) and Nature (1847 times). (C) Co-cited author network. Kim J was the most cited author (264 times), followed by Junn E (249 times) and Hebert SS (211 times).

Reference co-citation analysis revealed that 54,286 references were cited across the 1116 publications. The top 100 most frequently co-cited references are presented in Figure 5A. Among them, the most highly co-cited reference was cited 194 times, with a total link strength of 2339. The co-cited journal network is illustrated in Figure 5B. The analysis identified PLoS One as the most frequently cited journal (2266 citations), followed by Proceedings of the National Academy of Sciences of the United States of America with 2203 citations, and Nature with 1847 citations. In the co-cited author analysis (Fig. 5C), Kim J ranked first (264 citations), followed by Junn E (249 citations) and Hebert SS (211 citations).

### 3.4. Keywords analysis

**Figure 6.**
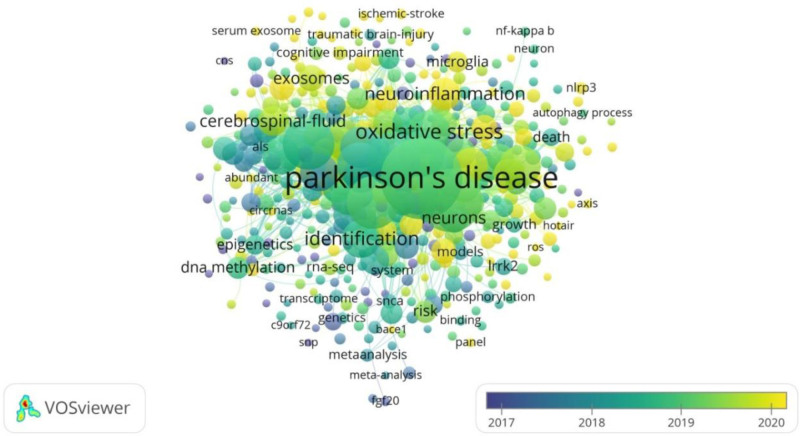
Co-occurrence analysis of keywords by overlay visualization. The figure presents a keyword co-occurrence network of Parkinson disease research from 2017 to 2020, generated using VOSviewer. Each node represents a keyword, with node size indicating its frequency of occurrence, and node color representing the average publication year (ranging from purple for earlier years to yellow for more recent years). Lines between nodes indicate co-occurrence relationships. High-frequency core keywords such as “Parkinson disease,” “oxidative stress,” and “neuroinflammation” reflect the main research hotspots and their evolving trends in this field.

VOSviewer was utilized to generate an overlay visualization map of keywords in this research field (Fig. 6). For this analysis, the minimum occurrence threshold for a keyword was set to 5. Out of 4490 initially identified keywords, 435 met this criterion. The top 10 keywords with the highest frequency of occurrence over the past decade are listed in Table 5. “PD” (509 occurrences), “expression” (306), “alpha-synuclein” (209), “Alzheimer disease” (200), and “miRNAs” (158) were the most frequently used keywords, each appearing more than 150 times.

**Table 5:** Top 10 co-occurrence keywords via VOSviewer.

Rank	Keywords	Records
1	Parkinson disease	509
2	Expression	306
3	Alpha-synuclein	209
4	Alzheimers disease	200
5	MicroRNAs	158
6	Oxidative stress	140
7	Neurodegeneration	128
8	Apoptosis	127
*9*	Biomarkers	123
10	Brain	97

VOSviewer analysis reveals “Parkinson disease,” “expression,” and “alpha-synuclein” as the most frequent keywords in the top 10, underscoring major research trends.

**Figure 7.**
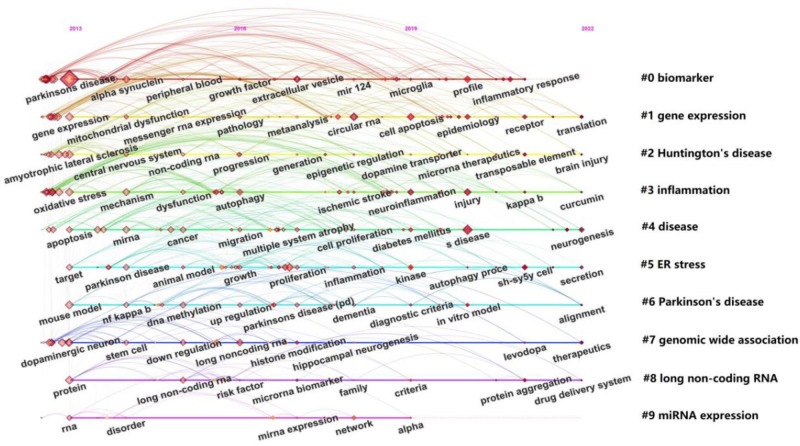
CiteSpace visualization map of timeline viewer. The figure displays the temporal evolution of major research topics from 2013 to 2022. Each horizontal line represents a keyword cluster, labeled on the right (#0–#9) according to their cluster ID and thematic content, including biomarker (#0), gene expression (#1), Huntington disease (#2), inflammation (#3), disease (#4), ER stress (#5), Parkinson disease (#6), genomic wide association (#7), long non-coding RNA (#8), and miRNA expression (#9). Nodes represent keywords, with their size indicating the frequency of occurrence, and colors representing the year of appearance (from yellow in earlier years to dark red in recent years). Links between nodes indicate co-occurrence relationships, and red tree-rings highlight keywords with significant citation bursts. This visualization reveals shifts in research hotspots and the emergence of new themes over time. miRNA = MicroRNA.

**Figure 8.**
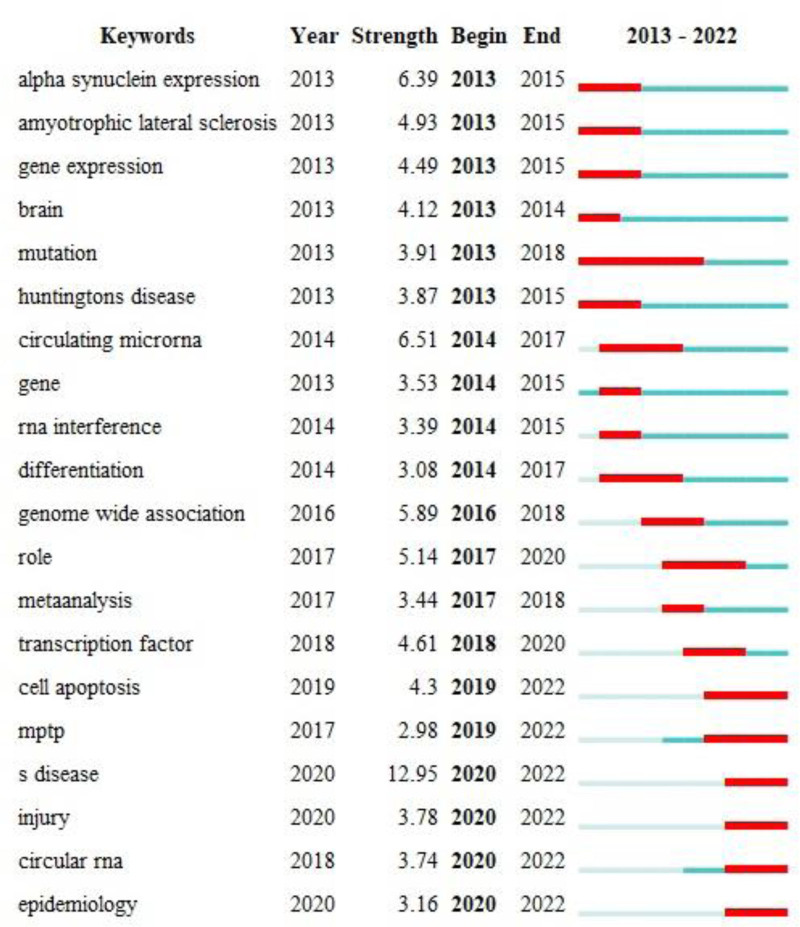
The top 20 keywords with the strongest citation bursts in this field. Red bars indicate periods of citation bursts. Early burst terms include “alpha synuclein expression” and “gene expression,” whereas recent burst terms such as “cell apoptosis” and “circular RNA” reflect emerging research hotspots.

Keyword cluster analysis and burst detection were also performed using CiteSpace to identify research hotspots and frontiers in PD and ncRNAs. The cluster analysis yielded 10 distinct clusters (Table 6), and Figure 7 presents a timeline view of keywords from the publications. Additionally, the top 20 keywords with the strongest citation bursts are displayed in Figure 8.

**Table 6:** The clusters in the field of PD and ncRNAs.

Cluster ID	Size	Silhouette	Mean (year)	Lable
0	59	0.699	2016	Biomarker
1	56	0.603	2017	Gene expression
2	51	0.638	2016	Huntington’s disease
3	48	0.71	2016	Inflammation
4	46	0.639	2017	Disease
5	46	0.729	2017	ER stress
6	40	0.661	2017	Parkinson disease
7	31	0.806	2015	Genome wide association
8	26	0.635	2017	Long non-coding RNA
9	15	0.836	2015	miRNA expression

The dominant research themes in Parkinson disease and ncRNA studies, as identified by keyword clustering, encompass “biomarker,” “gene expression,” and “Huntington disease,” with their temporal trends indicated by average emergence years.

ncRNAs = non-coding RNAs, PD = Parkinson disease, miRNA = microRNA.

## 4. Discussion

Mounting evidence over the past decade has highlighted the significant role of ncRNAs in the pathogenesis and potential therapy of PD. This study employs bibliometric and visual analysis to provide a comprehensive overview of this research field, with the aim of identifying global hotspots and anticipating future trends.

### 4.1. Publication outputs and growth trend

A total of 1116 articles were included in this study. The annual number of publications showed a marked increase, with 72.6% of the articles published between 2018 and 2022, indicating that ncRNAs have become a research hotspot in the field of PD. Although some fluctuations were observed in the annual publication count, the number of citations consistently rose year by year, peaking at 7690 in 2022. These findings further reflect the sustained interest in this topic. Scholars have recognized the importance of epigenetic regulation by ncRNAs in the progression of PD, and substantial efforts have been dedicated to elucidating their functions and underlying mechanisms.

### 4.2. Co-authorship: countries, institutions, and authors

China contributed the highest number of publications on this topic over the past decade, accounting for nearly 45% of the total, which reflects the country’s strong commitment to advancing this field. However, when countries were re-ranked by centrality, China did not appear among the top 10. Furthermore, among the top 5 most productive countries, China also ranked lower than the United States in terms of H-index. Together, these findings suggest that while China has actively engaged in ncRNA research related to PD, there remains a need for more influential and in-depth studies in this area.

Although Germany produced fewer publications than China or the USA, it exhibited the highest centrality, indicating its pivotal role in advancing this field. The USA ranked second in both publication count and centrality, underscoring its substantial contributions to the research domain. Notably, the USA also demonstrated the most extensive international collaborations, which may have contributed to its high centrality. These findings highlight the importance of fostering international collaboration among leading countries to integrate research resources and findings, thereby advancing the field into a new era.

Chinese institutions demonstrated high productivity over the past decade. However, few Chinese institutions ranked among the top 10 when evaluated by centrality. In terms of centrality, the leading institutions were the German Center for Neurodegenerative Diseases (Germany), University of Freiburg (Germany), and Banner Sun Health Research Institute (USA). The research output of these institutions spans multiple areas, ranging from ncRNA biomarkers to specific mechanisms of ncRNAs in PD.

Among the most prolific authors in this field were Ghafouri-fard S, Ghaedi K, and Taheri M. Ghafouri-Fard S and Taheri M focused on evaluating the expression of ncRNAs in PD pathophysiology, identifying BDNF- and nuclear factor-kappa B (NF-κB)-associated lncRNAs as potential contributors to PD pathogenesis. Meanwhile, Ghaedi K and colleagues demonstrated that miR-193b and miR-376a are associated with PD and may serve as potential diagnostic biomarkers. The consistent output from these researchers suggests a promising and expanding body of research in this area in the near future.

### 4.3. Co-citation: references, journals, and authors

In the co-citation analysis of references, Kim J and Junn E emerged as leading contributors to the rapid advancement of ncRNA and PD research. An article published by Kim J et al, titled “A MicroRNA Feedback Circuit in Midbrain Dopamine Neurons,” was the most frequently co-cited reference. The study investigated the role of miRNAs in mammalian dopamine neurons and demonstrated that miR-133b regulates dopaminergic activity through a feedback interaction with Pitx3. These findings provide new insights into the fine‐tuning mechanisms of dopaminergic signaling and contribute to the exploration of novel therapeutic targets for PD.

The second most co-cited reference was a study by Junn E et al, published in Proceedings of the National Academy of Sciences of the United States of America.^[12]^ Since PD is characterized by the accumulation of α-synuclein in Lewy bodies, targeting this pathogenic protein represents a promising therapeutic strategy. The study demonstrated that miR-7 plays a critical role in repressing α-synuclein expression and mitigating its toxicity. Moreover, the fourth most frequently cited reference further revealed that both miR-7 and miR-153 can inhibit α-synuclein protein expression by binding to the 3′-untranslated region of α-synuclein mRNA.^[13]^ Together, these studies suggest the existence of an miR-7-centered regulatory network that suppresses α-synuclein expression.

Mitochondrial dysfunction is one of the putative mechanisms implicated in PD. As demonstrated by the third most frequently co-cited reference, published in Nature, miR-34b and miR-34c are associated with mitochondrial impairment in PD.^[14]^ This mechanism may represent a potential therapeutic target for disease-modifying interventions.

Despite rapid advances in this field, it is noteworthy that most of the top 10 co-cited references were related to miRNAs, while research on other ncRNAs – such as lncRNAs and circRNAs – remains limited. On one hand, the functional mechanisms of lncRNAs and circRNAs are more complex than those of miRNAs, requiring further time and experimentation to elucidate. On the other hand, studies on lncRNAs and circRNAs in PD are likely to become the next research hotspot in the field.

### 4.4. Global research trends of ncRNAs on Parkinson disease

Keywords co-occurrence analysis serves as an effective method for identifying current research hotspots and frontiers. Our results indicated that terms such as “expression,” “alpha-synuclein,” “oxidative stress,” “apoptosis,” “biomarkers,” and “brain” exhibited higher citation frequencies. Furthermore, the keywords were categorized into 10 distinct clusters. Through this analysis, we identified global research trends and hotspots, which primarily centered on “biomarker,” “gene expression,” “Huntington disease (HD),” “inflammation,” “ER stress,” “PD,” “genome-wide association,” “lncRNAs,” and “miRNA expression.”

The mechanisms linking ncRNAs to PD through α-synuclein have garnered increasing attention, with a growing number of ncRNAs identified as regulators of α-synuclein. In addition to miR-7 and miR-153, studies have shown that long intergenic noncoding RNA-p21 upregulates α-synuclein expression by sponging miR-1277-5p in PD, ultimately promoting cell apoptosis.^[15]^ Regarding other ncRNA types, further evidence indicates that the circSNCA-miR-7-α-synuclein network positively regulates α-synuclein levels and induces apoptosis, suggesting that ncRNAs may serve as potential novel therapeutic targets for PD.^[10]^

The “inflammation” cluster has drawn particular attention. As shown in the timeline view, this cluster includes keywords such as oxidative stress, mechanism, autophagy, NF-κB, and curcumin. Notably, curcumin is currently a keyword with strong citation burst. Previous studies have indicated that curcumin exerts neuroprotective effects in PD by suppressing intestinal inflammation in animal models.^[16]^ Furthermore, recent research has established ncRNAs as key regulators of NF-κB signaling in PD. For example, downregulation of lncRNA-small nucleolar RNA host gene 7 was shown to inhibit inflammation and oxidative stress through the miR-425-5p/TRAF5/NF-κB axis.^[17]^ These findings suggest that classical inflammatory pathways remain a key research focus in understanding the mechanistic role of ncRNAs in PD.

Our analysis also identifies ER stress as an emerging cluster strongly associated with ncRNA research in PD. A study by Thanjeem Begum ME et al linked the expression of specific miRNAs (e.g., xxx-m0073-3p, xxx-m0225-3p, xxx-m0088-3p, xxx-m0098-5p) to ER stress in an in vitro PD model.^[18]^ Another study reported that overexpression of miR-384-5p promotes ER stress by interacting with glucose-regulated protein 78, a key regulator of the ER stress response, suggesting that inhibition of miR-384-5p may represent a therapeutic strategy for PD.^[19]^ These multilayered interactions position ER stress-related ncRNAs as attractive targets for both mechanistic exploration and therapeutic intervention. Together, these findings reveal a regulatory network of ncRNAs modulating ER stress in PD that warrants further investigation.

In summary, research addressing these issues could uncover new mechanisms and reveal novel therapeutic targets. Further mechanistic studies should examine whether modulating specific ncRNAs – via loss- or gain-of-function approaches – alters unfolded protein response (UPR) activation, α-synuclein aggregation, and neuronal survival in human-relevant models. Therapeutic strategies may focus on developing oligonucleotide-based agents, such as miRNA mimics or inhibitors, antisense oligonucleotides (ASOs), and LNA GapmeRs, as well as small molecules that restore proteostasis by normalizing ncRNA-regulated UPR signaling. Key challenges for clinical translation include achieving central nervous system -specific delivery, avoiding excessive suppression of adaptive UPR responses, and establishing disease-stage specificity by distinguishing between early adaptive and late maladaptive UPR activation.

### 4.5. Identification of common pathways in neurodegenerative diseases

As indicated by the keyword analysis, HD and amyotrophic lateral sclerosis (ALS) are among the top-ranked categories, both of which belong to the broader group of neurodegenerative diseases. These disorders, including PD and Alzheimer disease (AD), share partially overlapping pathological mechanisms and genetic factors.

A growing body of evidence suggests that certain genes are implicated across multiple neurodegenerative diseases. Recabarren D et al highlighted the potential involvement of PI3K-Akt signaling and thirteen miRNAs in ALS, AD, and PD.^[20]^ Another study performed RNA-seq analysis using peripheral blood mononuclear cells and identified dysregulated mRNAs and lncRNAs common to ALS, AD, and PD.^[21]^ Moreover, a gene expression microarray study conducted in brain tissues revealed several inflammation-related genes shared among these disorders.^[22]^ At the lncRNA level, NEAT1 has been reported to play a crucial role in AD, PD, HD, and ALS, while MALAT1 appears to function in AD and PD.^[23]^ These findings point to potential common ncRNA-related mechanisms underlying neurodegenerative diseases, offering new perspectives for research and therapeutic targeting.

### 4.6. Advancing lncRNA research in PD

In contrast to the more established roles of miRNAs, the functional landscape of lncRNAs in PD remains largely unexplored and presents unique challenges and opportunities. Although dysregulated lncRNAs have been preliminarily reported in PD blood or brain samples, their roles in core disease mechanisms have not been rigorously investigated. The primary hurdle lies in elucidating their complex and diverse mechanisms of action, which include epigenetic regulation, chromatin remodeling, and serving as competing endogenous RNAs. Future research should prioritize the use of single-cell RNA sequencing to map the cell-type-specific expression patterns of lncRNAs in vulnerable neuronal populations within the PD brain. Mechanistic studies must move beyond correlation and employ sophisticated tools such as CRISPR-based lncRNA knockout/knockdown and overexpression models in human-relevant cell cultures and animal models to definitively establish causality. Given their nuclear localization and structural versatility, lncRNAs like NEAT1 and MALAT1 are particularly promising targets for modulating broader transcriptional programs involved in neuroinflammation and mitochondrial function. The development of techniques to specifically target these lncRNAs, for instance using ASOs designed to disrupt their interactions with protein partners or DNA, will be crucial for translating these findings into novel therapeutic strategies.

### 4.7. Exploring the role of circular RNAs in PD

“Long non-coding RNA” and “miRNA expression” were identified as distinct clusters in the keyword analysis, whereas “circRNA” did not emerge as a separate cluster, reflecting the relatively limited understanding of its association with PD. CircRNAs possess 2 notable translational advantages: high stability and detectability in body fluids and exosomes, making them promising peripheral biomarkers. Functionally, circRNAs can regulate miRNA availability through the competing endogenous RNA mechanism, interact with proteins to modulate signaling complexes, and in some cases, be translated into functional peptides.

The first study linking circRNA to brain development was published in 2013.^[24]^ Since then, circRNAs have been increasingly associated with neurodegenerative diseases, including PD. Subsequent research has explored circRNA expression patterns in relation to PD prognosis and diagnosis using blood samples and postmortem tissues, leading to the identification of several potential biomarkers.^[9,25,26]^ A recent study by Cheng Q et al reported dysregulation of circSV2b in a PD mouse model.^[27]^ Mechanistic analysis revealed that circSV2b interacts with the miR-5107-5p–Foxk1–Akt1 axis, thereby regulating oxidative stress in PD. Other circRNAs, such as hsa_circ_0004381 and circTLK1, have also been shown to participate in dopaminergic neuron injury by modulating apoptosis.^[28,29]^ Therefore, investigating the role of circRNAs in PD represents an important direction for future research.

To advance the clinical translation of circRNA research, future efforts should focus on longitudinal human studies that measure circRNA levels in plasma, cerebrospinal fluid, or exosomal fractions. Such investigations are essential for evaluating the diagnostic and prognostic potential of circRNAs, as well as their dynamic changes across disease stages. Mechanistic studies should employ circRNA overexpression vectors, along with ASOs or small interfering RNAs targeting back-splice junctions, to establish causal roles of specific circRNAs in PD.

### 4.8. Advantages and shortcomings

This study presents the first comprehensive overview of ncRNA research in PD using bibliometric and visual analysis, offering insights into global hotspots and future trends. However, several limitations should be noted. First, the data were sourced exclusively from English articles available in the WoSCC, which may have led to the omission of relevant studies. Second, despite standardization efforts, potential biases may remain due to ambiguities in author names (e.g., authors sharing identical short names) and variations in keyword expression.

## 5. Conclusion

In summary, research on PD and ncRNAs has flourished over the past decade. China has demonstrated high productivity in terms of publication output, yet there is a need to strengthen international collaboration. Germany and the USA also play critical roles in advancing this field. Ghafouri-fard S, Ghaedi K, and Taheri M are among the most influential authors. Key emerging research hotspots include ER stress, inflammation, and circRNAs. This study provides scholars with a systematic overview of current trends and focal points in ncRNA and PD research.

## Author contributions

**Conceptualization:** Lina Zhu.

**Data curation:** Lina Zhu, Xiangxiu Wang, Chengqi He.

**Formal analysis:** Lina Zhu.

**Funding acquisition:** Lina Zhu, Deng Chen.

**Methodology:** Deng Chen.

**Project administration:** Chengqi He.

**Software:** Deng Chen.

**Writing – original draft:** Lina Zhu.

**Writing – review & editing:** Deng Chen, Xiangxiu Wang, Chengqi He.
